# Metabonomics Study of the Therapeutic Mechanism of Gynostemma pentaphyllum and Atorvastatin for Hyperlipidemia in Rats

**DOI:** 10.1371/journal.pone.0078731

**Published:** 2013-11-01

**Authors:** Miao Wang, Fei Wang, Yinan Wang, Xiaonan Ma, Min Zhao, Chunjie Zhao

**Affiliations:** 1 School of Pharmacy, Shenyang Pharmaceutical University, Shenyang, China; 2 College of Information Sci. and Eng., Northeastern University, Shenyang, China; 3 School of Life Science and Biopharmaceutics, Shenyang Pharmaceutical University, Shenyang, China; Boston University School of Medicine, United States of America

## Abstract

Gynostemma pentaphyllum (GP) is widely used for the treatment of diseases such as hyperlipidemia, fatty liver and obesity in China, and atorvastatin is broadly used as an anti-hyperlipidemia drug. This research focuses on the plasma and liver metabolites in the following four groups of rats: control, a hyperlipidemia model, a hyperlipidemia model treated with GP and a hyperlipidemia model treated with atorvastatin. Using ^1^H-NMR-based metabonomics, we elucidated the therapeutic mechanisms of GP and atorvastatin. Orthogonal Partial Least Squares-Discriminant analysis (OPLS-DA) plotting of the metabolic state and analysis of potential biomarkers in the plasma and liver correlated well with the results of biochemical assays. GP can effectively affect lipid metabolism, and it exerts its anti-hyperlipidemia effect by elevating the level of phosphatidylcholine and decreasing the level of trimethylamine N-oxide (TMAO). In contrast, atorvastatin affects hyperlipidemia mainly during lipid metabolism and protein metabolism *in vivo*.

## Introduction

Hyperlipidemia is a systemic disease that impairs the body in a generally unnoticeable, gradual, progressive and systemic way. The direct damage of hyperlipidemia can accelerate systemic arteriosclerosis, and it is an important risk factor for many diseases, such as stroke, coronary artery disease, myocardial infarction and cardiac sudden death [1]. Therefore, treating hyperlipidemia at its early stages is critical. In the clinic, combinations of statins and lipid traditional Chinese medicines are commonly used to treat hyperlipidemia in China.

Gynostemma pentaphyllum (GP) is easily accessible in China. It is a trailing plant that belongs to the cucurbitaceae family, and it mainly contains polysaccharides, flavones, saponins and trace elements [2-5]. Research has demonstrated the protective effects of gypenosides against fatty liver disease induced by a high fat and cholesterol diet and alcohol in rats [6,7]. GP promotes weight loss by regulating fat metabolism without causing side effects, such as diarrhea or altered appetite [8]. However, the mechanism by which GP combats hyperlipidemia is unknown. Atorvastatin belongs to the statin drug family, although it is an HMG-CoA reductase inhibitor [9], and its effect on other substances in the upstream and downstream metabolic pathways is unclear.

Metabonomics is regarded as another major research focus in addition the fields of genomics and the proteomics. It can provide complete biological system information at individual and group levels [10,11]. In the clinic, metabonomics can be used to prevent and diagnose diseases [12,13]. The discovery of specific biomarkers plays an important role in predicting disease progression, monitoring disease status after drug treatment and especially in clarifying of the mechanisms of action for traditional Chinese medicines [14]. Metabonomics is widely used to diagnose hyperlipidemia [15,16], monitor drug treatment of hyperlipidemia [17-20] and develop drugs to treat hyperlipidemia [21]. Compared with other methodologies, Nuclear Magnetic Resonance (NMR) spectrum is widely used as an unbiased metabonomics approach due to its high specificity, high resolution, sample preservation and its ability to analyze intact biological organization [22-24].

The focus of this research was on the plasma and liver metabolites of four groups of rats: a control group, a hyperlipidemia model group, a hyperlipidemia model group treated with GP and a hyperlipidemia group treated with atorvastatin. Here, the applicability of NMR-based metabonomics in assessing the effects of GP and atorvastain on hyperlipidemia in rats was evaluated to identify potential biomarkers and reveal the mechanisms of action for both drugs. By comparing the GP and atorvastatin treated groups, we determined the material basis, mechanism and action target of GP and atorvastain, revealing the mechanisms by which both drugs affect hyperlipidemia. 

**Figure 1:**
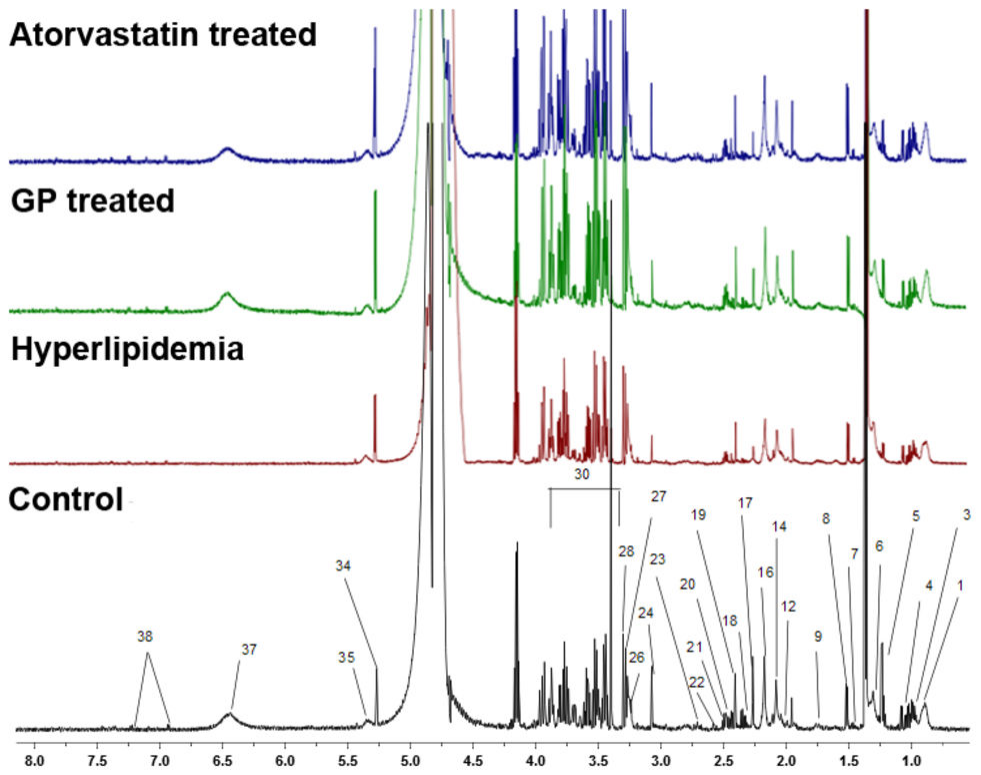
Typical 600 MHz ^1^H-NMR spectra of rat plasma samples. 1.Lipids (VLDL/LDL) 3. Isoleucine 4. Valine 5.3-Hydroxybutyrate 6. Lactate 7. Alanine 8. Lysine 9. Arginine 12. N-Acetyl glycoproteins 14. Glutamate 16. Acetoacetate 17. Acetone 18. Succinate 19. Pyruvate 20. Glutamine 21. Citrate 22. Glutathione 23. Aspartate 24. Creatine 26. Choline 27. Phosphocholine/GPC 28. TMAO 30. Glucose/aminoacids resonances 34.α-Glucose 35. Glycogen 37. Fumarate 38. Tyrosine.

## Materials and Methods

### Chemicals

Deuteroxide and sodium 3-trimethylsilyl-propionate [2,2,3,3,d4] (TSP) were purchased from Merck Drugs & Biotechnology (Germany). Dipotassium phosphate was obtained from Xilong Chemical Co., LTD. (Guangdong, China). Sodium hydroxide was purchased from Bodi Chemical co., LTD. (Tianjin, China). Distilled water was produced with a Milli-Q Reagent Water System.

**Figure 2:**
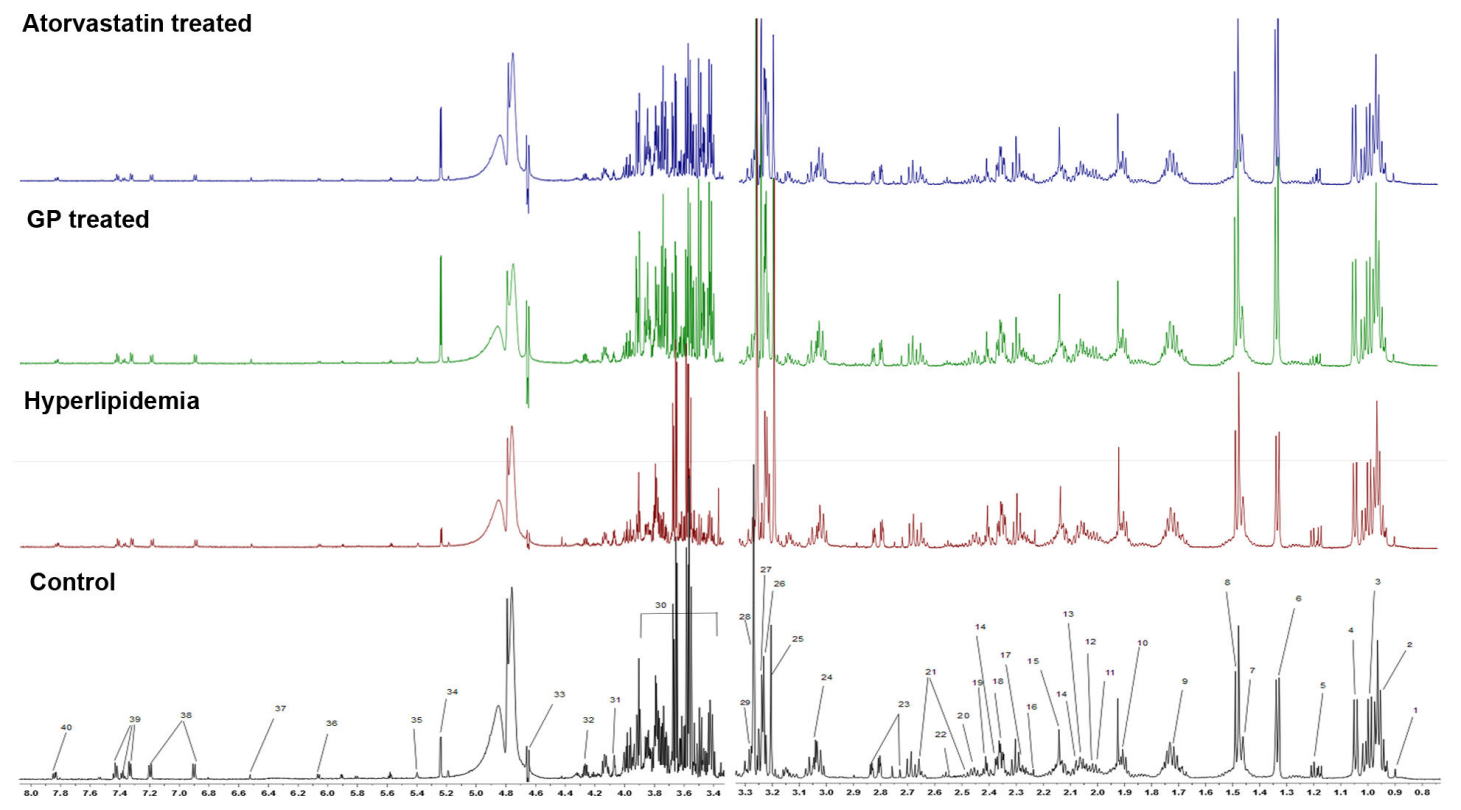
Typical 600 MHz ^1^H-NMR spectra of rat liver samples. 1. Lipids (VLDL/LDL) 2. Leucine 3. Isoleucine 4. Valine 5.3-Hydroxybutyrate 6. Lactate 7. Alanine 8. Lysine 9. Arginine 10. Acetate 11. Proline 12. N-Acetyl glycoproteins 13. O-Acetyl glycoproteins 14. Glutamate 15. Methionine 16. Acetoacetate 17. Acetone 18. Succinate 19. Pyruvate 20. Glutamine 21. Citrate 22. Glutathione 23. Aspartate 24. Creatine 25. Phosphatidylcholine 26. Choline 27. Phosphocholine/GPC 28. TMAO 29. Taurine 30. Glucose/aminoacids resonances 31.myo–Inositol 32. Threonine 33. β-Glucose 34.α-Glucose 35. Glycogen 36. Adenosine/Inosine 37. Fumarate 38. Tyrosine 39. Phenylalanine 40. Histidine.

### Ethics statement

All of the protocols using in this study were approved by the Medical Ethic Committee of Shenyang Pharmaceutical University, and all of the procedures were performed in accordance with the Regulations for the Administration of Affairs Concerning Experimental Animals and were approved by the State Council of People’s Republic of China. 

### Care and maintenance of animals

Male rats (Sprague Dawley, 6–8weeks, weighing 180–220 g, qualified number: SCXK (LIAO) 2010-0001) were obtained from Experimental Animal Center of Shenyang Pharmaceutical University (China). The rats were maintained under standard laboratory conditions (temperature, 23 ±2°C; relative humidity, 45–65%; and a natural day/night cycle) with food and water freely available.

### Construction of the hyperlipidemia model

After one week of adaptive breeding, the rats were randomly divided into four groups: a normal control group, a hyperlipidemia model group, a GP treatment group and an atorvastatin treatment group (n = 8 rats/group). With the exception of the control group, all of the groups were fed a high-fat diet that consisting of normal diet containing 15% lard, 5% yolk powder, 2% cholesterol, 1% sodium cholate and 0.2% propylthiouracil (Beijing Huafukang co., LTD.). The rats were bred continuously for eleven weeks. At 8:00 am weekly, 1.5 mL of blood was via retro-orbital bleeding from each rat under fasting conditions (water freely for 12 h). The blood samples were centrifuged at 4,000 × g for 10 min at 4 °C, and the supernatant was sent to Shenyang Red Cross Hospital to test levels of total cholesterol (TC), triglycerides (TG), high-density lipoprotein (HDL-C) and low density lipoprotein (LDL-C) to validate the model.

### Drug treatment

After the model was established, the four groups were fed a normal diet for four weeks. The rats in the GP treatment group were administered GP at a dose of 120 mg•Kg^-1^•d^-1^ (Guangzhou Baiyunshan Heji Huangpu Traditional Chinese Medicine Co., Ltd., batch number D1D001). The rats in the atorvastatin treatment group were administered atorvastatin calcium tablets at a dose of 1.8 mg•Kg^-1^•d^-1^ (Pfizer Pharmaceuticals Limited, batch number 018312k). The animals in the control and model groups received the same volume of normal saline as the drug-treated groups, and all of the administrations were performed by oral gavage.

### Sample collection for NMR analysis

#### Plasma collection

Two and a half milliliters of blood was collected from rats in each of the four groups at weeks 0, 11, and 15. The blood samples were collected in heparinized tubes, centrifuged at 4,000 x g for 15 min, and the supernatant was collected. The supernatants were stored at -80 °C until analysis.

#### Liver collection

After administration, rats in each of the four groups were sacrificed with ether, and the livers were collected. Liver homogenization was performed up by mixing 5 g of liver with 6 ml of saline, and the homogenates were stored at -80 °C until analysis.

### ^1^H-NMR spectroscopy of plasma

Three hundred and fifty microliters of plasma was transferred to EP tubes, and 300 μl of NaH_2_PO_4_-Na_2_HPO_4_ (0.2 M, pH7.4) buffer solution was added. The mixture was vortexed for 30 seconds and, centrifuged for 10 minutes at 13,000 x g; 550 μl of supernatant was obtained. Then, 150 μl TSP D_2_O solution (1.8 mg·ml^-1^) was added. The solution was vortexed for 30 seconds and transferred into a 5 mm NMR tube. The Carr-Purcell-Meiboom-Gill (CPMG) spectra were recorded.

### ^1^H-NMR spectroscopy of liver

Eight hundred microliters of liver homogenate were centrifuged for 10 minutes at 13,000 g, and 300 μl of the resulting supernatant was transferred into an EP tube. Three hundred microliters of buffer solution was added and the solution was then vortexed for 30 seconds and centrifuged for 10 minutes at 13,000 g. Five hundred microliters of the supernatant was transferred into an EP tube, and 150 μl TSP D_2_O solution (1.0 mg·ml^-1^) was added. Then, the solution was vortexed for 30 seconds and transferred to a 5 mm NMR tube. The CPMG spectra were recorded.

### ^1^H-NMR spectroscopy state

A superconducting Fourier transform nuclear magnetic resonance spectrometer of Brucker AV 600 MHz was used for the analyses. At a temperature of 298.2 K, CPMG was adopted, and the water peak was inhibited using the pre-saturation method. Sixty-four transients were collected, with 64 thousand data points for each spectrum and a spectral width of 12019 Hz and a pre-saturation delay of 2.0 s. The one dimensional NMR spectrum was obtained using the free induction decay signal by Fourier transform. 

**Table 1:** Plasma lipid levels of rats from the four groups. (x±s, n=8, mmol/L).

**Group**		**Week**	**TG**	**TC**	**HDL-C**	**LDL-C**
Control	0		0.87±0.08	1.42±0.17	0.69±0.06	0.17±0.03
	11		0.50±0.09	1.28±0.17	0.89±0.10	0.18±0.06
	15		0.94±0.16	1.17±0.07	0.91±0.13	0.17±0.02
Hyperlipidemia	0		0.89±0.13	1.65±0.09	0.70±0.06	0.17±0.04
	11		0.87±0.12	6.12±1.86a**	0.68±0.18	1.12±0.74 a**
	15		1.15±0.11	3.78±0.22	0.98±0.09	0.72±0.15
GP treated	0		1.02±0.26	1.48±0.22	0.69±0.06	0.18±0.03
	11		0.90±0.13	6.34±1.19 a**	0.68±0.12	1.21±0.14 a**
	15		0.87±0.18	1.83±0.12 b**	0.94±0.06	0.55±0.25 b**
Atorvastatin treated	0		1.23±0.28	1.42±0.15	0.71±0.08	0.16±0.03
	11		1.24±0.39	6.22±1.03 a**	0.63±0.57	1.18±0.23 a**
	15		0.92±0.16	2.00±0.31 b**	0.94±0.20	0.59±0.16 b**

a: compared with normal, b: compared with model within group;* *P*<0.05, ** *P*<0.01

### Data reduction of NMR spectra and pattern recognition

All the collected samples were analyzed with the validated method, and all the results obtained by biochemical analyses were expressed as the means ± standard deviation in each group. Each sample was represented by an NMR graph. The acquired NMR spectra were referenced to the chemical shift of TSP (chemical shift δ0.0 ppm). Following phase and baseline correction, the ^1^H-NMR spectra were automatically reduced to TXT files using MestReNova 5.3.1 (Mestrelab Research, USA). This applications manager incorporates a peak deconvolution package that allows for the detection of the chemical shift and area of the peaks eluted from each spectrogram. The data from each spectrum (over the range of 0–10 ppm) was reduced and normalized to the total of all the resonance integral regions. The regions containing the resonance from residual water (4.60–5.16 ppm) were excluded [25]. Each reduced bucket had an equal width of 0.04 ppm, and each integral region was effectively standardized to a ratio of the total metabolites detected in the sample. The TXT files were imported into Microsoft Excel for labeling, and then imported into SIMCA-P 12.0 (Umetrics, Umea, Sweden) for principal component analysis. Prior to the analyses, the values of all of the variables were centered and scaled. For the identification of potential markers, we used the HMDB database (http://www.hmdb.ca/).

PCA (Principal Component Analysis) was applied to identify outliers and detect data grouping or separation trends, and it also produced an overview of the data set. From the score and loading plots, we classified the samples and the potential biomarkers responsible for the classification are shown. The supervised pattern recognition, OPLS-DA, focused on the actual class discriminating variation of data compared to the unsupervised approach, PCA [26]. The OPLS-DA model was validated by describing R^2^Y and Q^2^ values. Q^2^ was used to provide an estimation of the predictive capability of the models, whereas R^2^ describes how well the data could be mathematically reproduced by the training model. The fact that both Q^2^Y and R^2^Y were close to 1 indicates that it is an excellent model, whereas the poor ratio of these values suggests model overfitting [27,28]. Some of the intensities from the spectral data of key metabolites selected according to variable importance plot (VIP) analysis of OPLS were expressed as the means ± sd. The significance of variation between groups in the data regarding biological parameters was determined using the paired-sample t-test, using SPSS19.0 (IBM, USA). *P*-values of less than 0.05 were considered to be statistically significant.

**Figure 3:**
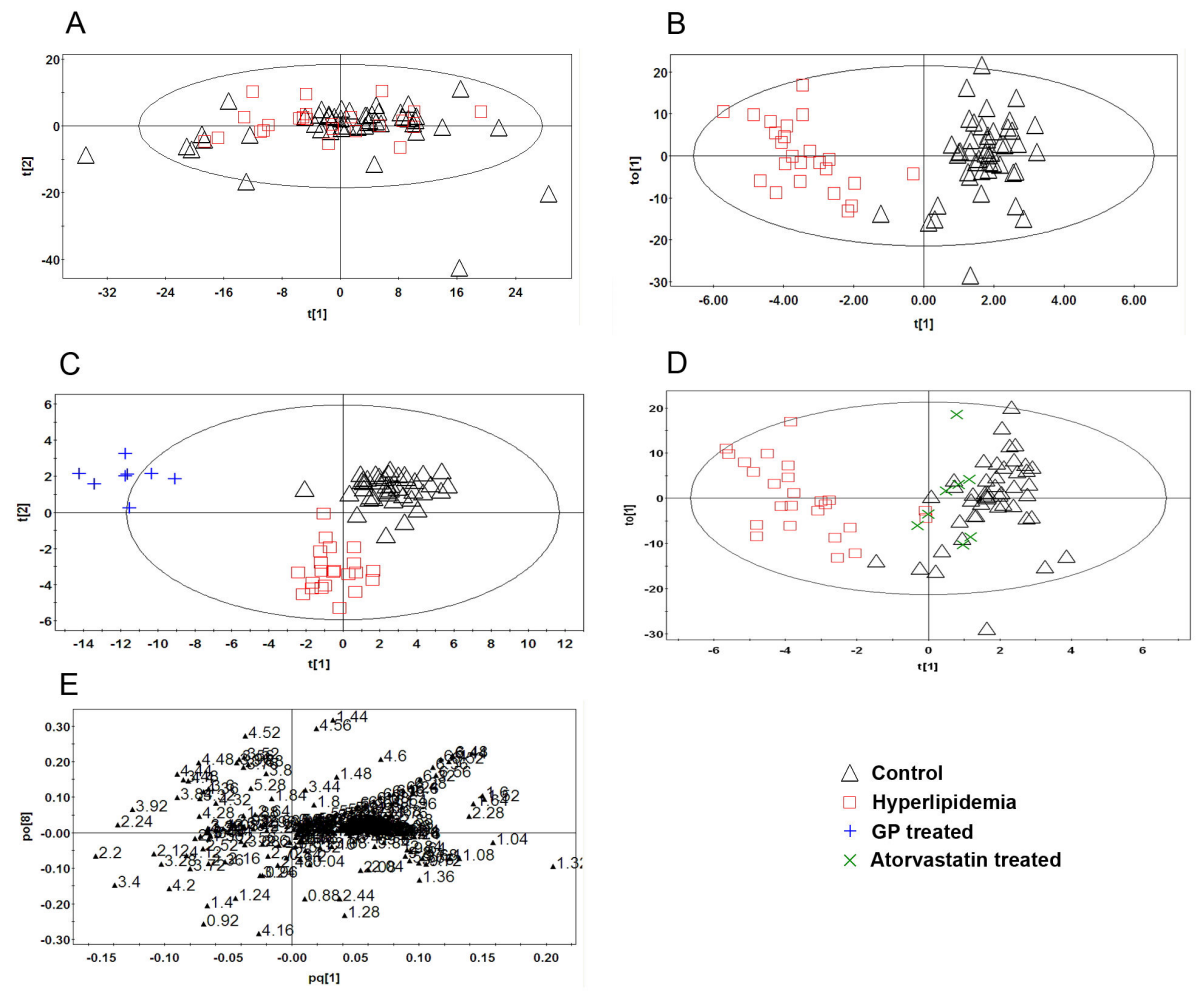
PR analysis of the ^1^H-NMR spectra of rat plasma. (A): PCA analysis of the spectra of plasma from normal and hyperlipidemia rats (R^2^X=0.988, Q^2^=0.885). (B): Scores plot of the OPLS-DA analysis of the spectra from the plasma of normal and hyperlipidemia rats (R^2^X=0.925, R^2^Y=0.874, Q^2^=0.642). (C): Scores plot of the OPLS-DA analysis of the spectra from the plasma of normal, hyperlipidemia and GP-treated rats (R^2^X=0.968, R^2^Y=0.878, Q^2^=0.538). (D): Scores plot of the OPLS-DA analysis of the spectra from the plasma of normal, hyperlipidemia and Atorvastatin-treated rats (R^2^X=0.909, R^2^Y=0.522, Q^2^=0.328). (E): Loading plot of the OPLS-DA analysis of the spectra from the plasma of normal and hyperlipidemia rats.

## Results

### TC, TG, LDL-C, and HDL-C test results

The plasma lipid levels of the rats from the four different groups are shown in Table 1. The variations of TC, TG LDL-C and HDL-C in rats from the control group were not significant, and neither were the variations observed in the other 3 groups in the first 6 weeks. However, from the 7th week on, the levels of TC and LDL-C gradually increased, and remarkable differences appeared by week 11 (*P*<0.01), indicating that our model successfully resembled hyperlipidemia in rats.

**Figure 4:**
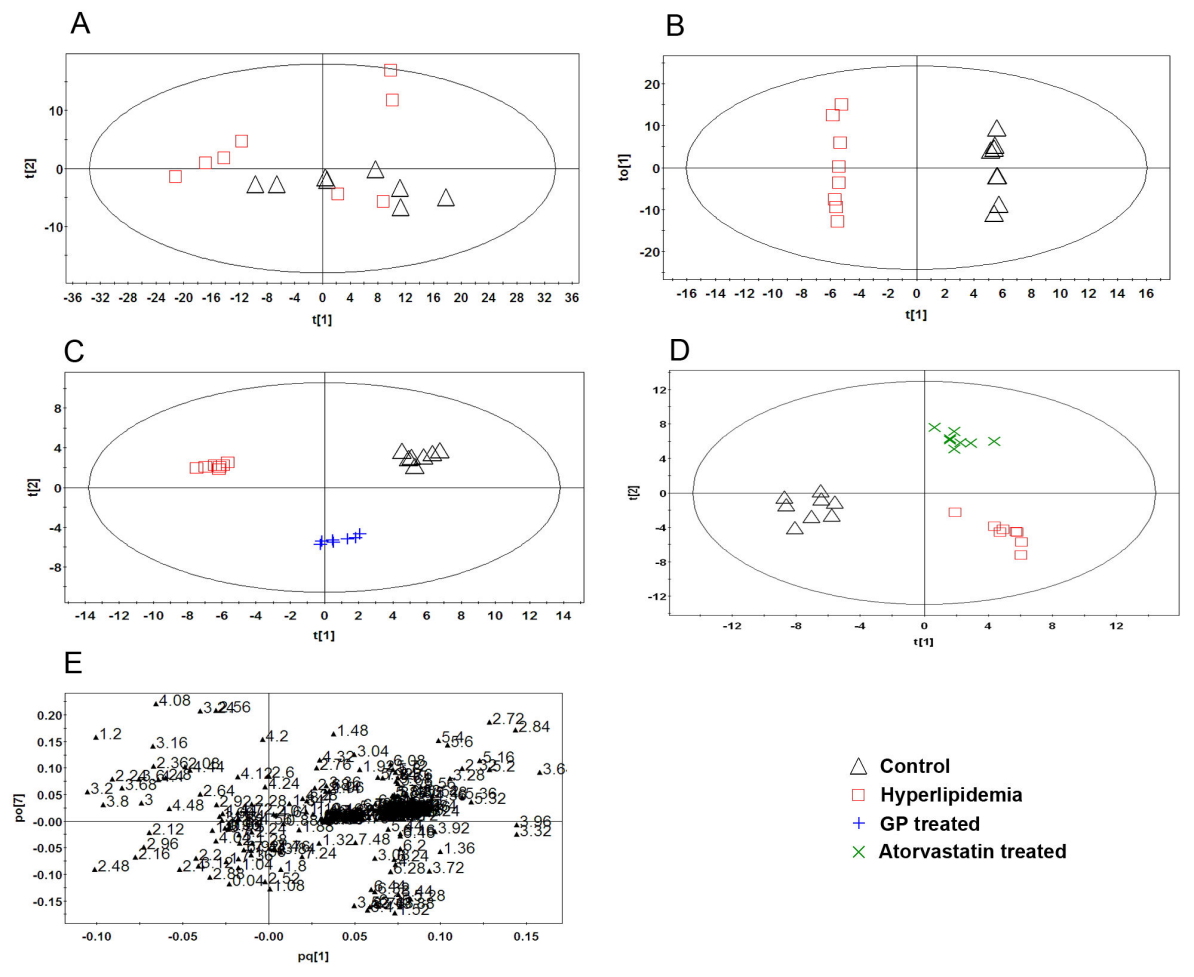
PR analysis of ^1^H-NMR spectra of rat liver tissues. (A): PCA analysis of the spectra of liver tissues from normal and hyperlipidemia rats (R^2^X=0.955, Q^2^=0.782). (B): Scores plot of the OPLS-DA analysis of the spectra from the liver tissues of normal and hyperlipidemia rats (R^2^X=0.953, R^2^Y=0.999, Q^2^=0.827). (C): Scores plot of the OPLS-DA analysis of the spectra from the liver tissues of normal, hyperlipidemia and GP-treated rats (R^2^X=0.955, R^2^Y=0.984, Q^2^=0.608). (D): Scores plot of the OPLS-DA analysis of the spectra from the liver tissues of normal, hyperlipidemia and Atorvastatin-treated rats (R^2^X=0.931, R^2^Y=0.945, Q^2^=0.544). (E): Loading plot of the OPLS-DA analysis of the spectra from the liver tissues of normal and hyperlipidemia rats.

After one week of gavage with GP and atorvastatin, the levels of TC and LDL-C began to decrease gradually, and after 3 weeks, the reduction in TC and LDL-C levels was highly significant (*P*<0.01), suggesting that both drugs could lower lipid levels.

**Table 2:** Relative integrals from selected metabolites that contributed to the classification of the rats in the four groups.

**Biological matrices**	**Metabolites**	**Control**	**Hyperlipidemia**	**GP**	**Atorvastatin**	**Changes (H-C)**	**P-value (H-C)**	**Changes (G-H)**	**P-value (G-H)**	**Changes (A-H)**	**P-value (A-H)**
**Plasma**	Acetoacetate	233.1±53.8	283.1±68.0	233.0±43.0	256.9±31.9	↑	0.03	—	0.683	—	0.812
	Acetone	13.4±3.5	18.8±4.1	12.6±2.9	14.9±3.6	↑	0.001	↓	0.017	—	0. 374
	Valine	23.5±6.9	17.7±4.8	17.8±4.1	24.4±3.1	↓	0.005	—	0.862	↑	0.004
	Isoleucine	105.8±26.4	90.8±16.0	91.5±22.1	115.5±11.3	↓	0.011	—	0.395	↑	0.001
	Alanine	64.0±18.8	49.5±8.7	51.4±13.7	65.5±9.3	↓	0.010	—	0.776	↑	0.008
	3-HB	38.4±17.6	27.3±9.2	23.5±11.4	34.9±9.4	↓	0.009	—	0.564	↑	0.023
	Lactate	229.2±66.5	179.1±29.1	166.4±39.9	172.3±35.3	↓	0.011	—	0.290	—	0.644
	Lysine	64.0±18.8	49.4±10.4	51.4±13.7	63.9±17.4	↓	0.011	—	0.938	—	0.155
	TMAO	254.5±54.7	295.7±39.6	235.0±51.2	302.7±45.2	↑	0.012	↓	0.002	—	0.725
	Fumarate	12.9±5.5	9.19±3.8	8.63±2.8	14.7±2.6	↓	0.005	—	0.221	↑	0.035
**Liver**	Acetoacetate	131.6±8.7	174.6±37.1	139.2±27.8	127.8±14.6	↑	0.013	↓	0.047	↓	0.008
	Acetone	19.3±1.4	24.1±4.5	19.6±2.4	18.2±2.8	↑	0.025	↓	0.015	↓	0.010
	Glutamine	131.6±8.72	63.0±11.1	13.4±8.4	45.0±9.2	↑	0.013	↓	0.047	↓	0.008
	Fumarate	8.11±2.3	5.31±3.3	6.41± 3.4	8.28±5.7	↓	0.045	—	0.519	—	0.287
	Phosphatidylcholine	34.3±4.0	24.7±5.1	31.7±2.4	28.3±9.9	↓	0.004	↑	0.020	—	0.429
	Glycogen	12.0±0.9	7.86±4.2	6.83±5.4	8.23±3.5	↓	0.042	—	0.726	—	0.843
	Citrate	43.9±2.2	35.8±5.3	37.5±6.9	33.6±2.9	↓	0.004	—	0.692	—	0.289

The data were normalized to the total of all the resonance integral regions over the range of 0.04–10.0 ppm, excluding the resonance from residual water (4.60–5.16 ppm).

“↑” and “↓ ” indicated that the compound was up- and down-regulated, “— ”indicated that the compound did not significantly change;

P-values determined using paired-sample t-test, and *P*-values of less than 0.05 were considered to be statistically significant. H-C, G-H and A-H represented hyperlipidemia vs. control, GP vs. hyperlipidemia and atorvastatin vs. hyperlipidemia respectively.

### Qualitative 1H-NMR analysis and identification

By applying ^1^H-NMR, which was previously used to study bio-fluid, we obtained a spectrogram of the metabolite components from one dimension high resolution *H spectrum* [29,30]. The ^1^H- NMR spectrums of the plasma and liver of rats in the four different groups are shown in Figure 1 and Figure 2. The chemical shift and splitting of the main metabolites in the spectra were identified by referencing published literature [15,31-33]. The identification of metabolites in the plasma and liver of rats is shown in Table S1.

**Figure 5:**
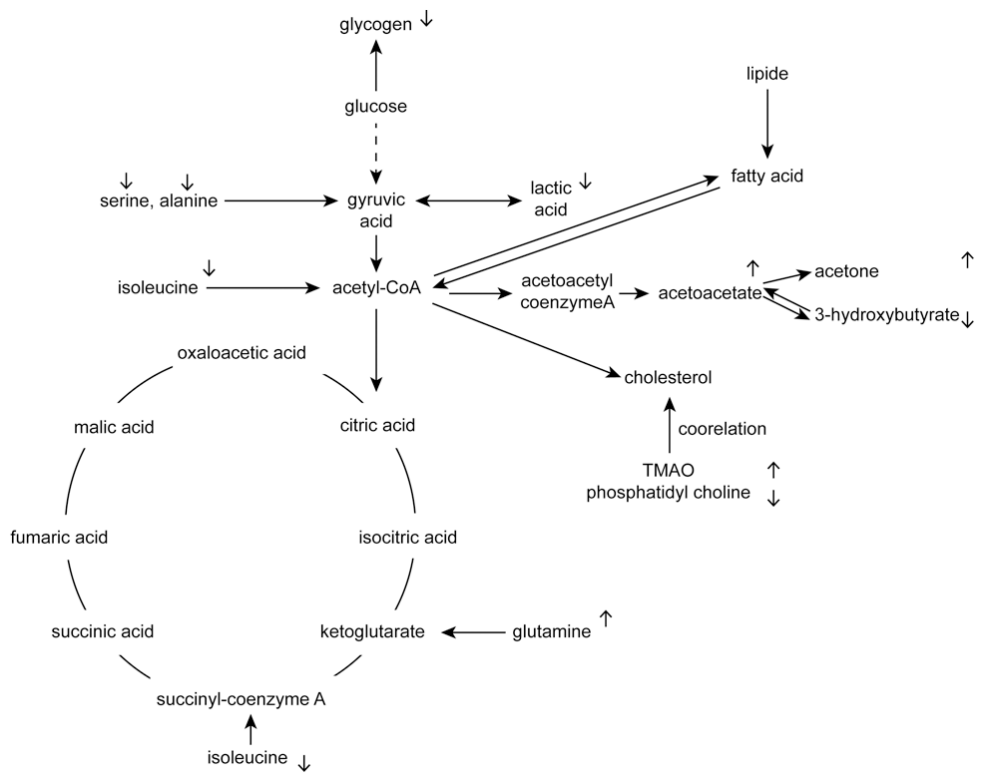
Summary of the metabolic pathways related to the metabolites that changed significantly in the hyperlipidemia model. “↑” and “↓” indicate that the compound is up- and down-regulated compared with the control group.

### Multivariate analysis of potential biomarkers

#### Metabolic changes in the plasma

After centralization and normalization of integral data using SIMCA-P12.0, pattern recognition analysis was conducted by employing PCA and OPLS-DA. In Figure 3, each point represents one sample, and different sample assembly revealed different metabolic patterns. 

PCA analysis of the ^1^H-NMR spectra of plasma from rats in the control group and hyperlipidemia model group are shown in Figure 3A. Although the samples from two groups could be separated from each other, the classification was not remarkable. We therefore employed a supervised learning method to remove non-essential factors to improve the accuracy of classification. In this study, the OPLS-DA algorithm was employed to discriminate between the normal and hyperlipidemia model groups (Figure 3B). The OPLS-DA model had a high R^2^Y value (0.92) and Q^2^ value (0.63), indicating the overall goodness of fit and good predictive capabilities of the proposed model. The results of the control, hyperlipidemia model and GP/atorvastatin treatment groups are shown in Figure 3C and 3D. The control and hyperlipidemia model groups were clearly discriminated due to remarkable differences between their metabolic profiles. The GP and atorvastatin treatment groups were distributed between the control and hyperlipidemia model groups, suggesting that the metabolic profiles of the hyperlipidemia rats recovered and the plasma metabolites were restored to normal levels after drug treatment.

Potential biomarkers were selected according to the VIP values from the pattern recognition model. In the OPLS model, 80 variables displayed VIP values greater than 1.2. In the loading plot (as shown in Figure 3E), these 80 points were relatively far away from the dense cluster, suggesting that these samples provided a greater contribution to the classification. Points whose VIP values were greater than 1.2 and with *P* values less than 0.05 were regarded as final biomarkers. Following structural identification, 10 potential biomarkers were identified, as listed in Table 2.

Compared with the normal control group, the levels of acetoacetic (δ2.22 ppm), acetone (δ2.27 ppm) and TMAO (δ3.26 ppm) increased in the hyperlipidemia model group, while the levels of valine (δ1.06 ppm), isoleucine (δ0.98 ppm), alanine (δ1.48 ppm), 3- hydroxybutyrate (δ1.2 ppm), lactate (δ1.34 ppm), lysine (δ1.5 ppm) and fumarate (δ6.52 ppm) levels decreased. The variation between biomarkers in the hyperlipidemia model group and the GP and atorvastatin treatment groups is shown in Table 2, and the results of other metabolites in the plasma are listed in Table S2.

#### Metabolic changes in the liver

The PCA and OPLS-DA of the ^1^H-NMR spectra from rat livers in the control and hyperlipidemia model control groups are shown in Figure 4A and Figure 4B, respectively. The OPLS-DA model had a high R^2^Y value (0.991) and Q^2^ value (0.685), indicating the overall goodness of fit and good predictive capabilities of the model. 

The OPLS-DA results from the normal, hyperlipidemia model and the GP treatment groups are shown in Figure 4C. The results of the normal, hyperlipidemia model and atorvastatin treatment groups are shown in Figure 4D. The control and hyperlipidemia model groups were clearly segregated due to the remarkable difference between their metabolic profiles. The GP and atorvastatin treatment groups were distributed between the control and hyperlipidemia model groups, indicating that the metabolic profile of hyperlipidemia rats was repaired and the liver metabolites were restored to normal levels after drug treatment. The loading plot is shown in Figure 4E.

Potential biomarkers were selected according to the VIP values from the pattern recognition model. In the OPLS model, 40 variables displayed VIP values were greater than 1.2. In the loading plot (Figure 4E), these 40 points are relatively far away from the dense cluster, suggesting that these samples provided a greater contribution to the classification. Points whose VIP values were greater than 1.2 with *P* values less than 0.05 were regarded as final biomarkers. Following structural identification, 7 potential biomarkers were identified, as listed in Table 2.

Compared with the control group, the levels of acetoacetic (δ2.22 ppm), acetone (δ2.27 ppm) and glutamine (δ2.46 ppm) increased in the hyperlipidemia model group, while fumarate (δ6.52 ppm), glycogen (δ5.4 ppm), phosphatidylcholine (δ3.22 ppm) and citrate (δ2.5 ppm) levels decreased. The variation between biomarkers in the hyperlipidemia model group and the GP and atorvastatin treatment groups is shown in Table 2, and the results of other liver metabolites are listed in Table S3.

## Discussion

This study demonstrated that the levels of glutamine, acetone, TMAO and acetoacetate increased dramatically, while the levels of valine, isoleucine, alanine, 3-hydroxybutyrate, lactate, lysine, fumarate, glycogen, phosphatidylcholine and citrate decreased remarkably in the plasma and liver of hyperlipidemia model rats compared to the control group. These data suggest that hyperlipidemia is closely related to carbohydrate metabolism, lipid metabolism and amino acid metabolism *in vivo* [34].

Lactate, fumarate, citrate and glycogen are involved in carbohydrate metabolism. The decrease in lactate indicates a change in glucose metabolic pathways, resulting in enhanced aerobic metabolism or lipid synthesis. The decrease in fumarate and citrate levels, which are the intermediate products of tricarboxylic acid cycle, indicates the deregulation of energy metabolism. Meanwhile, these data indirectly show that the decrease in lactate will shift the glucose metabolic pathway to lipid synthesis. The decrease in glycogen indicates that glycogenesis is inhibited or glycogenolysis is enhanced.

3-hydroxybutyrate, acetoacetate, acetone, TMAO and phosphatidylcholine are related to lipid metabolism. 3-hydroxybutyrate, acetone and acetoacetate are generally considered to be ketone bodies. Our data indicate that the levels of acetoacetate and acetone increase, while the level of 3-hydroxybutyrate decreases in the plasma and liver of hyperlipidemia model rats. Thus, hyperlipidemia can lead to the accumulation of ketone bodies. Acetoacetate is produced by acetyl-CoA, and it then generates 3-hydroxybutyrate or acetone. The decrease in 3-hydroxybutyrate demonstrates that the conversion of acetoacetate transfers towards the production of acetone. It was previously reported that TMAO in plasma is produced by the following pathway: dietary phosphatidylcholine/choline → gut flora-formed TMA → hepatic FMO-formed TMAO; TMAO is positively related to hyperlipidemia and atherosclerosis [35]. In this research, high-fat diet enriched phosphatidylcholine was used to induce hyperlipidemia, demonstrating that a high-fat diet can increase TMAO levels in the plasma. Phosphatidylcholine can affect the deposition of lipids and cholesterol by removing excessive triglycerides and improving the solubility of cholesterol and lipids in the plasma. Furthermore, it has antioxidant properties.  Hyperlipidemia can decrease phosphatidylcholine levels.

Alanine, isoleucine, valine, lysine and glutamine are related to protein metabolism. Alanine, isoleucine and valine are glucogenic amino acids. The decrease in these amino acids indicates that they generated α-keto acid through deamination, and α-keto acid subsequently generates glucose via gluconeogenesis. The decrease in the ketogenic amino acid lysine indicates it is transformed to generate ketone bodies or fatty acids, which could indirectly explain the accumulation of ketone bodies. Glutamine can transport ammonia, as it is the mechanism of ammonia transportation and storage. The decrease in glucogenic amino acids implies that with oxidative decomposition of amino acids, the resulting ammonia combines with glutamate and can be transported as glutamine, increasing glutamine levels. 

The discovery of biomarkers and the biological explanations mentioned above can be used to analyze the pathogenesis of hyperlipidemia through metabolic pathways, and these results can likewise play an important role in assisting the clinical diagnose of hyperlipidemia (Figure 5). 

The levels of acetoacetate, acetone and glutamine in the GP and atorvastatin treatment groups decreased remarkably prior to drug administration, and they returned to normal levels after administration. The variations of lactate, lysine, glycogen and citrate were not significant. The level of phosphatidylcholine increased remarkably, while TMAO levels decreased significantly after GP treatment; no changes were observed in these markers in response to atorvastatin treatment. In contrast, the levels of valine, isoleucine, alanine, fumarate and 3-hydroxybutyrate increased significantly after atorvastatin treatment, while there were no significant changes in these markers after GP treatment.

In summary, GP acts by affecting lipid metabolism. After hyperlipidemia model rats were treated with GP, cholesterol and low density lipoprotein levels in the plasma decreased and ketone body metabolites returned to normal, but only a weak effect was observed on protein and glucose metabolism. In contrast to the mechanism of atorvastatin, GP was able to return phosphatidylcholine and TMAO levels to normal. Phosphatidylcholine consists of hydrophobic nonpolar groups and hydrophilic polar groups that have strong surface activity and emulsification. Phosphatidylcholine can improve the absorption and utilization of lipids, decrease the retention of lipids in vessels, remove cholesterol deposits in blood vessel walls, promote diffusion of atherosclerotic spots and reduce high cholesterol. The unsaturated fatty acid of molecules can prevent the absorption of cholesterol in the intestinum tenue and promote cholesterol excretion [36]. Meanwhile, TMAO is closely related to phosphatidylcholine metabolism. The ability of GP to decrease in TMAO suggests that GP has an inhibitory effect on the pathway of phosphatidylcholine to TMAO.

Atorvastatin acts mainly via the pathways of lipid metabolism and protein metabolism. After hyperlipidemia model rats were bred with atorvastatin, HMG-CoA reductase was competitively inhibited in the liver, decreasing cholesterol synthesis and increasing low-density lipoprotein receptor synthesis. As a result, levels of low-density lipoprotein cholesterol decreased. This effect can have preventive and therapeutic effects on arteriosclerosis and coronary heart disease. Metabolic analyses of the plasma and liver indicate that atorvastatin restored the level of ketone bodies, acetoacetate, acetone and 3-hydroxybutyrate to normal. Moreover, it affected protein metabolism, which returned glucogenic amino acid and glutamine levels to normal but had a weak effect on glucose metabolism.

In this study, ^1^H-NMR metabonomics combined with PCA and OPLS-DA were used to analyze metabolite profiles. Through the comparative study of atorvastatin, the mechanisms of two drugs were revealed according to the variations of endogenous metabolites, providing scientific evidence for the application of metabonomics for the mechanism study of traditional Chinese medicine.

## Supporting Information

Table S1 **^1^H Chemical shift assignment of the metabolites in plasma and liver of rats.**
(DOC)

Table S2 **Relative integrals from all metabolites except biomarkers in the plasma of rats.**
(DOC)

Table S3 **Relative integrals from all metabolites except biomarkers in the liver of rats.**
(DOC)

## References

[1] Chen HZ (2004) Current status of blood lipid level and treatment of hyperlipoidemia in Chinese population. Journal of Chinese integrative medicine. Mar 2(2):81-2. 15339461 10.3736/jcim20040201

[2] Li N, Wu CF, Xu XY, Liu ZY, Li X et al. (2012) Triterpenes possessing an unprecedented skeleton isolated from hydrolyzate of total saponins from Gynostemma pentaphyllum. Eur J Med Chem 50: 173-178. doi:10.1016/j.ejmech.2012.01.052. PubMed: 22342101. 22342101

[3] Kao TH, Huang SC, Inbaraj BS, Chen BH (2008) Determination of flavonoids and saponins in Gynostemma pentaphyllum (Thunb.) Makino by liquid chromatography-mass spectrometry. Anal Chim Acta.626(2): 200-211. doi:10.1016/j.aca.2008.07.049. PubMed: 18790122. 18790122

[4] Liang SX, Sun HW (2002) Determination of six nutritional elements in Chinese herbal medicines by graphite furnace atomic absorption spectrometry. SPECTROSCOPY Spectral Anal. 22(5): 847-849. 12938448

[5] Yang X, Zhao Y, Yang Y, Ruan Y (2008) Isolation and characterization of immunostimulatory polysaccharide from an herb tea, Gynostemma pentaphyllum Makino. J Agric Food Chem 56(16): 6905-6909. doi:10.1021/jf801101u. PubMed: 18636735. 18636735

[6] Huyen VT, Phan DV, Thang P, Ky PT, Hoa NK et al. (2012) Antidiabetic Effects of Add-On Gynostemma pentaphyllum Extract Therapy with Sulfonylureas in Type 2 Diabetic Patients. Complement Alternat Med, 2012: 2012:452313. PubMed: 23125867 10.1155/2012/452313 PMC3484409 23125867

[7] Qin R, Zhang J, Li C, Zhang X, Xiong A et al. (7 2012) Protective effects of gypenosides against fatty liver disease induced by high fat and cholesterol diet and alcohol in rats. Arch Pharm Res 7;35(7): 1241-1250. doi:10.1007/s12272-012-0715-5. PubMed: 22864747. 22864747

[8] la Cour B, Mølgaard P, Yi Z (1995) Traditional Chinese medicine in treatment of hyperlipidaemia. J Ethnopharmacol 46(2): 125-129. doi:10.1016/0378-8741(95)01234-5. PubMed: 7650951. 7650951

[9] Naoumova RP, Dunn S, Rallidis L, Abu-Muhana O, Neuwirth C et al. ( 7 1997) Prolonged inhibition of cholesterol synthesis explains the efficacy of atorvastatin. J Lipid Res 7;38(7): 1496-1500. PubMed: 9254075. 9254075

[10] Watkins SM, German JB (2002) Metabolomics and biochemical profiling in drug discovery and development. Curr Opin Mol Ther Jun;4(3): 224-228. PubMed: 12139307. 12139307

[11] Wishart DS (2007) Current Progress in computational metabolomics. Brief Bioinform. 8: 279–293. doi:10.1093/bib/bbm030. PubMed: 17626065. 17626065

[12] Hollywood K, Brison DR, Goodacre R (2006) Metabolomics: Current technologies and future trends. Proteomics. 6: 4716–4723. doi:10.1002/pmic.200600106. PubMed: 16888765. 16888765

[13] Lindon JC, Holmes E, Nicholson JK (2004) Metabonomics and its role in drug development and disease diagnostics. Expert Rev Mol Diagn 4: 189–199. doi:10.1586/14737159.4.2.189. PubMed: 14995905. 14995905

[14] Zhang A, Sun H, Wang X (. 8 2012) Recent highlights of metabolomics for traditional Chinese medicine. Pharmazie. 8;67(8): 667-675. PubMed: 22957430. 22957430

[15] Zhang Q, Wang GJ, Jiye A, Ma B, Dua Y et al. (2010) Metabonomic profiling of diet-induced hyperlipidaemia in a rat model. Biomarkers. 15(3): 205–216. doi:10.3109/13547500903419049. PubMed: 20001218. 20001218

[16] Jean CM, Cécile C , Bernadette D, Genevieve A, Denis L et al. (2009) H NMR metabonomics can differentiate the early atherogenic effect of dairy products in hyperlipidemic hamsters1. Atherosclerosis. 206: 127–133. doi:10.1016/j.atherosclerosis.2009.01.040. PubMed: 19324361. 19324361

[17] Zha WB, Jiye A, Wang GJ, Yan B, Gu SH et al. (2009) Metabonomic characterization of early atherosclerosis in hamsters with induced cholesterol .Biomarkers. 14(6): 372–380. doi:10.1080/13547500903026401. PubMed: 19552617. 19552617

[18] Zhang Q, Wang GJ, Jiye A , Wu D, Zhu LL et al. (2009) Application of GC/MS-based metabonomic profiling in studying the lipid-regulating effects of Ginkgo biloba extract on diet-induced hyperlipidemia in rats. Acta Pharmacol Sin. 30: 1674–1687. doi:10.1038/aps.2009.173. PubMed: 19960012. 19960012 PMC4077396

[19] Gu Y, Zhang YF, Shi XZ, Li XY , Hong J et al. (2010) Effect of traditional Chinese medicine berberine on type 2 diabetes based on comprehensive metabonomics. Talanta. 81: 766–772. doi:10.1016/j.talanta.2010.01.015. PubMed: 20298851. 20298851

[20] Liu F, Gan PP , Wu HN , Woo WS, Eng SO et al. (2012) A combination of metabolomics and metallomics studies of urine and serum from hypercholesterolaemic rats after berberine injection. Anal Bioanal Chem 403: 847–856. doi:10.1007/s00216-012-5923-9. PubMed: 22434276. 22434276

[21] Sun Y, Lian ZQ, Jiang CY, Wang YH, Zhu HB (2012) Beneficial Metabolic Effects of 29,39,59-tri-acetyl-N6- (3-Hydroxylaniline) Adenosine in the Liver and Plasma of Hyperlipidemic Hamsters. PLOS ONE. 7(3): e32115. doi:10.1371/journal.pone.0032115. PubMed: 22470419. 22470419 PMC3314636

[22] Beckonert O, Keun HC, Ebbels TMD, Bundy JG, Holmes E et al. (2007) Metabolic profiling, metabolomics and metabonomic procedures for NMR spectroscopy of urine, plasma, serum and tissue extracts. Nat Protoc 2: 2692–2703. doi:10.1038/nprot.2007.376. PubMed: 18007604. 18007604

[23] Azmi J, Connelly J, Holmes E, Nicholson JK, Shore RF et al. (2005) Characterization of the biochemical effects of 1- nitronaphthalene in rats using global metabolic profiling by NMR spectroscopy and pattern recognition. Biomarkers. 10: 401–416. doi:10.1080/13547500500309259. PubMed: 16308265. 16308265

[24] Nicholson JK, Lindon JC, Holmes E (1999) ‘Metabonomics’: understanding themetabolic responses of living systems to pathophysiological stimuli viamultivariate statistical analysis of biological NMR spectroscopic data. Xenobiotica. 29: 1181–1189. doi:10.1080/004982599238047. PubMed: 10598751. 10598751

[25] Wei L, Liao P, Wu H, Li X, Pei F et al. (2008) Toxicological effects of cinnabar in rats by NMR-based metabolic profiling of urine and serum. Toxicol Appl Pharmacol 227: 417–429. doi:10.1016/j.taap.2007.11.015. PubMed: 18164359. 18164359

[26] Gavaghan CL, Wilson ID, Nicholson JK (2002) Physiological variation in metabolic phenotyping and functional genomic studies: use of orthogonal signal correction and PLS-DA. FEBS Lett 530: 191–196. doi:10.1016/S0014-5793(02)03476-2. PubMed: 12387891. 12387891

[27] Liang X, Chen X, Liang Q, Zhang H, Hu P et al. (2011) Metabonomic study of chinese medicine Shuanglong formula as an effective treatment for myocardial infarction in rats. J Proteome Res 10: 790–799. doi:10.1021/pr1009299. PubMed: 21090666. 21090666

[28] Barker M, Rayens W (2003) Partial least squares for discrimination. J Chemom. 17: 166–173. doi:10.1002/cem.785.

[29] Bales JR, Higham DP, Howe I, Nicholson JK, Sadler PJ (1984) Use of high-resolution proton nuclear magnetic resonance spectroscopy for rapid multi-component analysis of urine. Clin Chem 30(4): 426–432. PubMed: 6321058. 6321058

[30] Wishart DS, Lewis MJ, Morrissey JA, Flegel MD, Jeroncic K et al. (2008) The human cerebrospinal fluid metabolome. J Chromatogr B Anal Technol Biomed Life Sci 871: 164–173. doi:10.1016/j.jchromb.2008.05.001. PubMed: 18502700. 18502700

[31] Fan WM (1996) Metabolite profiling by one- and two-dimensional NMR analysis of complex mixtures. Prog Nucl Magn Reson Spectrosc 28: 161–219. doi:10.1016/0079-6565(95)01017-3.

[32] Bollard ME, Garrod S, Holmes E, Lincoln JC, Humpfer E et al. (2000) Highresolution 1H and 1H-13C magic angle spinning NMR spectroscopy of rat liver. Magn Reson Med 44: 201–207. doi:10.1002/1522-2594(200008)44:2. PubMed: 10918318. 10918318

[33] He Q, Ren P, Kong X, Wu Y, Wu G et al. ( 2 2012) Comparison of serum metabolite compositions between obese and lean growing pigs using an NMR-based metabonomic approach. J Nutr Biochem 2;23(2): 133-139. doi:10.1016/j.jnutbio.2010.11.007. PubMed: 21429726. 21429726

[34] Donald V, Judith GV, Pratt Charlotte W (2000) Fundamentals of Biochemistry. John Wiley & Sons, Inc.

[35] Wang Z, Klipfell E, Bennett BJ et al (2011) Gut flora metabolism of phosphatidylcholine promotes cardiovascular disease. Nature;472: 57–63. doi:10.1038/nature09922. PubMed: 21475195. 21475195 PMC3086762

[36] Gibellini F, Smith TK (2010) The Kennedy pathway—denovo synthesis of phosphatidylethanolamine and phosphatidylcholine. IUBMB Life. 62: 414–428. PubMed: 20503434. 20503434 10.1002/iub.337

