# Supplementary material for: Metabonomics Study of the Therapeutic Mechanism of Gynostemma pentaphyllum and Atorvastatin for Hyperlipidemia in Rats
Source: PLoS One. 2013 Nov 1;8(11):e78731. doi: 10.1371/journal.pone.0078731 (PMC3815346; doi:10.1371/journal.pone.0078731)
Supplement: Table S1 — 1H Chemical shift assignment of the metabolites in plasma and liver of rats. (DOC) [file pone.0078731.s001.doc]

**Table S1. ^1^H Chemical shift assignment of the metabolites in plasma and liver of rats**

| **NO.** | **Plasma** | | | **Liver** | | |
| --- | --- | --- | --- | --- | --- | --- |
|  | **Metabolites** | **Moieties** | **δ1H(ppm)and multiplicity** | **Metabolites** | **Moieties** | **δ^1^H(ppm)and multiplicity** |
| 1 | Lipids(VLDL/LDL) | CH3, -(CH2)n- | 0.9(t) | Lipids(VLDL/LDL) | CH3, -(CH2)n- | 0.9(t) |
| 2 | - | - | - | Leucine | γCH3 | 0.94(d) |
| 3 | Isoleucine | δCH3 | 0.98(d) | Isoleucine | δCH3 | 0.98(d) |
| 4 | Valine | γCH3 | 1.06(d) | Valine | γCH3 | 1.06(d) |
| 5 | 3-Hydroxybutyrate | γCH3 | 1.20(d) | 3-Hydroxybutyrate | γCH3 | 1.20(d) |
| 6 | Lactate | βCH3,αCH | 1.34(d),4.11(q) | Lactate | βCH3,αCH | 1.34(d),4.11(q) |
| 7 | Alanine | βCH3 | 1.48(d) | Alanine | βCH3 | 1.48(d) |
| 8 | Lysine | γCH2 | 1.5(m) | Lysine | γCH2 | 1.5(m) |
| 9 | Arginine | γCH2 | 1.73(m) | Arginine | γCH2 | 1.73(m) |
| 10 | - | - | - | Acetate | βCH3 | 1.91(s) |
| 11 | - | - | - | Proline | γCH2,δCH2,αCH | 2.00(m),3.35(t),4.11(t) |
| 12 | N-Acetyl glycoproteins | CH3 | 2.02(s) | N-Acetyl glycoproteins | CH3 | 2.02(s) |
| 13 | - | - | - | O-Acetyl glycoproteins | CH3 | 2.06(s) |
| 14 | Glutamate | βCH2, γCH2 | 2.08(m),2.38(m) | Glutamate | βCH2, γCH2 | 2.08(m),2.38(m) |
| 15 | - | - | - | Methionine | S-CH3 | 2.14(s) |
| 16 | Acetoacetate | CH3 | 2.22(s) | Acetoacetate | CH3 | 2.22(s) |
| 17 | Acetone | CH3 | 2.27(s) | Acetone | CH3 | 2.27(s) |
| 18 | Succinate | CH2 | 2.36(s) | Succinate | CH2 | 2.36(s) |
| 19 | Pyruvate | CH3 | 2.41(s) | Pyruvate | CH3 | 2.41(s) |
| 20 | Glutamine | γCH2 | 2.46(m) | Glutamine | γCH2 | 2.46(m) |
| 21 | Citrate | Half CH2,Half CH2 | 2.50(d)，2.66(d) | Citrate | Half CH2,Half CH2 | 2.50(d)，2.66(d) |
| 22 | Glutathione | γCH2, S-CH2 | 2.54(m),2.96(dd) | Glutathione | γCH2, S-CH2 | 2.54(m),2.96(dd) |
| 23 | Aspartate | βCH2, αCH | 2.72(dd),2.84(dd) 3.89(dd) | Aspartate | βCH2, αCH | 2.72(dd),2.84(dd),3.89(dd) |
| 24 | Creatine | N-CH3 | 3.06(s) | Creatine | N-CH3 | 3.06(s) |
| 25 | - | - | - | Phosphatidylcholine | N(CH3)3 | 3.22(s) |
| 26 | Choline | N(CH3)3 | 3.24(s) | Choline | N(CH3)3 | 3.24(s) |
| 27 | Phosphocholine/GPC | N(CH3)3 | 3.25(s) | Phosphocholine/GPC | N(CH3)3 | 3.25(s) |
| 28 | TMAO | N(CH3)3 | 3.26(s) | TMAO | N(CH3)3 | 3.26(s) |
| 29 | - | - | - | Taurine | N-CH2,S-CH2 | 3.27(t),3.44(t) |
| 30 | Glucose/aminoacids resonances | ringprotons/α-CH | 3.3-3.9 | Glucose/aminoacids resonances | ringprotons/α-CH | 3.3-3.9 |
| 31 | - | - | - | myo -Inositol | 2-CH | 4.06(t) |
| 32 | - | - | - | Threonine | γCH2 | 4.28(m) |
| 33 | - | - | - | β-Glucose | CH | 4.66(d) |
| 34 | α-Glucose | CH | 5.24(d) | α-Glucose | CH | 5.24(d) |
| 35 | Glycogen | CH | 5.4 | Glycogen | CH | 5.4 |
| 36 | - | - | - | Adenosine/Inosine | ring protons | 6.08(d) |
| 37 | Fumarate | CH= | 6.52(s) | Fumarate | CH= | 6.52(s) |
| 38 | Tyrosine | CH,CH | 6.87(m),7.20(m) | Tyrosine | CH,CH | 6.87(m),7.20(m) |
| 39 | - | - | - | Phenylalanine | 2,6-CH, 3,5-CH, 4-CH | 7.33(m), 7.38(m), 7.42(m) |
| 40 | - | - | - | Histidine | ring protons | 7.90(s) |

s=singlet; d=doublet; dd=double doublet; t=triplet; q=quartet; m=multiplet.
