# Supplementary material for: Metabonomics Study of the Therapeutic Mechanism of Gynostemma pentaphyllum and Atorvastatin for Hyperlipidemia in Rats
Source: PLoS One. 2013 Nov 1;8(11):e78731. doi: 10.1371/journal.pone.0078731 (PMC3815346; doi:10.1371/journal.pone.0078731)
Supplement: Table S2 — Relative integrals from all metabolites except biomarkers in the plasma of rats. (DOC) [file pone.0078731.s002.doc]

**Table S2. Relative integrals from all metabolites except biomarkers in the plasma of rats** .

| **Metabolites** | **Control** | **Hyperlipidemia** | **GP** | **Atorvastatin** | ***P*-value** | | |
| --- | --- | --- | --- | --- | --- | --- | --- |
|  |  |  |  |  | **Hyperlipidemia vs Control** | **GP vs Hyperlipidemia** | **Atorvastatin vs Hyperlipidemia** |
| Arginine | 207.3±62.3 | 215.3±40.5 | 166.7±37.8 | 197.9±27.9 | 0.602 | 0.131 | 0.401 |
| N-Acetyl glycoproteins | 54.1±18 | 49.3±13 | 37.7±13 | 48.1±8.8 | 0.234 | 0.084 | 0.427 |
| Glutamate | 20.9±7.9 | 21.5±8.4 | 10.8±5.9 | 22.3±6.4 | 0.572 | 0.035 | 0.653 |
| Succinate | 24.6±13 | 30.0±11 | 19.6±13 | 34.1±8.3 | 0.250 | 0.362 | 0.119 |
| Pyruvate | 18.5±11 | 16.2±7.1 | 11.1±6.0 | 21.1±6.8 | 0.245 | 0.654 | 0.065 |
| Glutamine | 33.7±14 | 34.6±9.8 | 23.9±11 | 38.1±6.9 | 0.513 | 0.100 | 0.459 |
| Citrate | 6.92±4.3 | 7.14±5.5 | 9.72±6.6 | 6.43±4.9 | 0.861 | 0.709 | 0.662 |
| Glutathione | 275.1±85.0 | 141.6±50.1 | 347.6±52.6 | 258.4±31.6 | 0.328 | 0.728 | 0.998 |
| Aspartate | 215.9±61.6 | 226.0±48.3 | 173.8±39.7 | 216.3±31.7 | 0.755 | 0.313 | 0.621 |
| Creatine | 53.0±19 | 51.8±12 | 47.9±17 | 70.1±10 | 0.741 | 0.647 | 0.011 |
| Choline | 59.1±24 | 63.6±44 | 47.8±18 | 58.8±13 | 0.481 | 0.204 | 0.351 |
| Phosphocholine/GPC | 258.6±56.3 | 287.4±43.4 | 235.0±51.1 | 252.6±40.7 | 0.162 | 0.032 | 0.102 |
| α-Glucose | 32.3±20.1 | 36.1±22 | 40.9±18 | 35.0±26 | 0.748 | 0.173 | 0.778 |
| Glycogen | 21.5±13 | 14.4±9.5 | 15.0±8.3 | 16.4±9.5 | 0.511 | 0.152 | 0.923 |
| Tyrosine | 6.23±2.1 | 8.51±3.4 | 20.8±4.4 | 8.07±2.0 | 0.414 | 0.011 | 0.727 |

Datas were normalized to the total of all the resonance integral regions over the range of 0.04–10.0 ppm excluding the resonance from residual water (4.60–5.16 ppm);

P-values determined using paired-sample t-test, P-values less than 0.05 were considered significant.
