# Supplementary material for: Metabonomics Study of the Therapeutic Mechanism of Gynostemma pentaphyllum and Atorvastatin for Hyperlipidemia in Rats
Source: PLoS One. 2013 Nov 1;8(11):e78731. doi: 10.1371/journal.pone.0078731 (PMC3815346; doi:10.1371/journal.pone.0078731)
Supplement: Table S3 — Relative integrals from all metabolites except biomarkers in the liver of rats. (DOC) [file pone.0078731.s003.doc]

**Table S3. Relative integrals from all metabolites except biomarkers in the liver of rats** .

| **Metabolites** | **Control** | **Hyperlipidemia** | **GP** | **Atorvastatin** | ***P*-value** | | |
| --- | --- | --- | --- | --- | --- | --- | --- |
|  |  |  |  |  | **Hyperlipidemia vs Control** | **GP vs Hyperlipidemia** | **Atorvastatin vs Hyperlipidemia** |
| Leucine | 125.6±10.9 | 103.5±24.4 | 145.4±31.3 | 161.3±17.3 | 0.061 | 0.002 | 0.002 |
| Isoleucine | 202.6±15.1 | 217.2±75.5 | 205.6±75.1 | 171.2±33.4 | 0.584 | 0.761 | 0.127 |
| Valine | 164.6±8.87 | 170.7±46.3 | 164.5±52.1 | 132.9±16.3 | 0.712 | 0.813 | 0.046 |
| 3-Hydroxybutyrate | 57.1±2.2 | 57.8±8.6 | 55.9±8.3 | 48.0±6.5 | 0.823 | 0.567 | 0.030 |
| Lactate | 58.5±5.0 | 65.2±11 | 39.4±8.4 | 47.1±5.9 | 0.244 | 0.005 | 0.010 |
| Alanine | 249.9±19.9 | 237.0±39.4 | 241.0±40.2 | 224.9±32.5 | 0.510 | 0.760 | 0.515 |
| Lysine | 237.9±25.7 | 212.6±34.0 | 228.0±29.0 | 187.1±12.2 | 0.215 | 0.348 | 0.056 |
| Arginine | 245.4±29.0 | 212.9±62.7 | 363.0±73.6 | 389.6±58.1 | 0.269 | 0.001 | 0.001 |
| Proline | 38.0±1.7 | 36.9±3.9 | 38.6±3.8 | 37.0±3.5 | 0.378 | 0.388 | 0.898 |
| N-Acetyl glycoproteins | 87.6±3.0 | 87.3±11 | 80.9±13 | 71.5±6.7 | 0.956 | 0.200 | 0.007 |
| O-Acetyl glycoproteins | 121.2±5.76 | 126.7±13.1 | 113.6±21.7 | 99.8±7.6 | 0.362 | 0.050 | 0.001 |
| Succinate | 92.5±8.8 | 104.1±19.4 | 90.3±24 | 77.8±8.0 | 0.212 | 0.090 | 0.016 |
| Pyruvate | 97.6±7.8 | 90.6±10 | 100.9±19.0 | 85.6±8.0 | 0.219 | 0.348 | 0.377 |
| Glutathione | 7.61±2.4 | 11.2±5.3 | 10.6±5.2 | 7.02±3.8 | 0.148 | 0.882 | 0.038 |
| Aspartate | 7.77±2.1 | 9.85±3.8 | 6.78±2.4 | 6.21±3.99 | 0.212 | 0.030 | 0.073 |
| Creatine | 107.1±10.1 | 100.5±13.8 | 107.9±13.7 | 87.8±8.8 | 0.413 | 0.284 | 0.019 |
| Choline | 385.7±48.4 | 414.6±83.1 | 273.7±49.5 | 360.9±24.2 | 0.501 | 0.014 | 0.132 |
| Phosphocholine/GPC | 385.7±48.4 | 414.6±83.1 | 273.7±49.5 | 360.9±24.2 | 0.501 | 0.014 | 0.132 |
| TMAO | 476.0±36.8 | 414.3±103 | 470.8±129 | 456.1±79.4 | 0.179 | 0.428 | 0.366 |
| Taurine | 253.4±39.3 | 196.9±80.9 | 381.3±118 | 449.7±74.2 | 0.153 | 0.001 | 0.001 |
| myo -Inositol | 58.5±5.0 | 65.2±11 | 39.4±8.4 | 47.1±5.9 | 0.244 | 0.005 | 0.010 |
| Threonine | 52.6±2.0 | 51.3±8.1 | 51.2±11.7 | 43.6±5.3 | 0.642 | 0.976 | 0.040 |
| β-Glucose | 20.6±6.4 | 20.0±14 | 21.4±7.9 | 26.6±13 | 0.932 | 0.725 | 0.388 |
| α-Glucose | 33.8±5.1 | 23.1±12 | 45.8±13 | 59.5±9.5 | 0.103 | 0.001 | 0.001 |
| Adenosine/Inosine | 8.40±3.2 | 5.33±4.1 | 4.39±3.9 | 4.62±3.5 | 0.100 | 0.627 | 0.625 |
| Tyrosine | 17.3±1.4 | 18.7±10 | 15.3±9 | 12.6±3.4 | 0.705 | 0.518 | 0.127 |
| Phenylalanine | 44.4±4.0 | 47.0±19 | 41.1±21 | 36.7±8.3 | 0.697 | 0.625 | 0.178 |
| Histidine | 6.88±4.0 | 2.89±1.3 | 3.75±2.0 | 3.27±2.0 | 0.059 | 0.663 | 0.798 |

Datas were normalized to the total of all the resonance integral regions over the range of 0.04–10.0 ppm excluding the resonance from residual water (4.60–5.16 ppm);

P-values determined using paired-sample t-test, P-values less than 0.05 were considered significant.
